# Characterization and Antibacterial Evaluation of Biodegradable Mannose-Conjugated Fe-MIL-88NH_2_ Composites Containing Vancomycin against Methicillin-Resistant *Staphylococcus aureus* Strains

**DOI:** 10.3390/polym14132712

**Published:** 2022-07-01

**Authors:** Haseena, Muddaser Shah, Khadija Rehman, Adnan Khan, Arshad Farid, Carlotta Marini, Alessandro Di Cerbo, Muhammad Raza Shah

**Affiliations:** 1 Institute of Chemical Sciences, University of Peshawar, Peshawar 25120, Pakistan; haseenafarid5@gmail.com; 2 Department of Botany, Abdul Wali Khan University Mardan, Mardan 23200, Pakistan; 3 Natural and Medical Sciences Research Center, University of Nizwa, P.O. Box 33, Birkat Al Mauz, Nizwa 616, Oman; 4 International Centre for Chemical and Biological Sciences, Research Institute of Chemistry, University of Karachi, Karachi 74200, Pakistan; khadija.rehman@iccs.edu; 5 Gomal Center of Biochemistry and Biotechnology, Gomal University, Dera Ismail Khan 29050, Pakistan; arshadfarid@gu.edu.pk; 6 School of Biosciences and Veterinary Medicine, University of Camerino, 62024 Matelica, Italy; carlotta.marini@unicam.it (C.M.); alessandro.dicerbo@unicam.it (A.D.C.)

**Keywords:** metal-organic frameworks, vancomycin, multiple drug resistant, *Staphylococcus aureus*, mannose

## Abstract

The emergence of bacterial resistance has increased the economic burden of infectious diseases dramatically during the previous few decades. Multidrug resistance (MDR) is difficult to cure in both Gram-negative and positive bacteria and is often incurable with traditional and broad-range antibiotics. Therefore, developing techniques to increase the antibacterial activity of therapeutic drugs is essential. Metal-organic frameworks (MOFs) are extremely versatile hybrid materials made of metal ions coupled via organic bridging ligands. They have been widely used as an excellent vehicle for drug delivery due to their low toxicity, biodegradability, and structural stability upon loading and functionalization. The present study focused on the synthesis of mannose (MNS)-coated MOFs with enhanced surface contact with *S. aureus* cells. The MNS coating on the surface of MOFs enhances their adherence to bacteria by binding to lectins present on the bacterial cell, resulting in improved VCM cellular penetration and activity against resistant bacteria. Various techniques, including atomic force microscopy, DLS, TGA, FT-IR, and DSC, were employed to analyze MNS-coated MOFs. They were also evaluated for their efficacy against resistant *S. aureus*. The results indicated that when VCM was loaded into MNS-coated MOFs, their bactericidal activity rose dramatically, resulting in the greater suppression of resistant *S. aureus*. AFM investigation of *S. aureus* strains demonstrated total morphological distortion after treatment with MNS-coated drug-loaded MOFs. The results of this work suggest that MNS-coated MOFs may be effective for reversing bacterial resistance to VCM and open new pathways for improving antibiotic therapy for diseases associated with MDR.

## 1. Introduction

Antimicrobial resistance has now become a global issue due to its association with increased morbidity and mortality [1]. The emergence of bacterial resistance in the last few decades has increased significantly and has enhanced the economic burden of infectious diseases [2,3]. This resistance emerged because of certain factors of which the improper and excessive use of antibiotics is a leading cause [4,5,6,7,8,9,10,11,12,13,14]. Additionally, certain bacteria are capable of developing or acquiring resistance to various antimicrobial drugs; this is regarded as the multidrug resistance (MDR) [15]. MDR is difficult to treat in both Gram-positive and -negative bacteria and is often incurable with traditional and broad-spectrum antibiotics [1,16].

Moreover, the surviving capability of resistant bacteria is further increased upon treatment with antibiotics. For example, *Staphylococcus aureus* strains (40–60%) collected from hospitals in the United States show resistance to methicillin (MRSA, methicillin-resistant *Staphylococcus aureus*), and, in certain circumstances, even to last-resort antibiotics such as VCM and carbapenems [17]. Vancomycin (VCM) is a bactericidal glycopeptide antibiotic of the first generation that has been used in the treatment of certain infections caused by Gram-positive bacteria, such as MRSA. VCM kills the bacterial cell by inhibiting trans glycosylase function and cell wall synthesis via binding with cell wall precursors [18]. The emerging resistance reduced the susceptibility of VCM against *Staphylococcus aureus* because it limits the penetration of VCM into the bacterial cell [19].

Furthermore, resistance generated via alteration in peptidoglycan terminus inhibits VCM binding with the bacterial cell, which creates hurdles to prevent cell wall synthesis [19]. The first VCM-intermediate-resistant *Staphylococcus aureus* (VISA) was identified in the 1997 [20]. The treatment of these resistant strains necessitates a high dose, which results in increased toxicity, prolonged hospitalization, and an increase in bacterial mortality [21]. As a result, research and the scientific community have developed a larger interest in enhancing the antibacterial properties of the therapeutic drug. In all these circumstances, nanotechnology has been provided a better platform because of its high biocompatibility, low toxicity, low cost, and better drug delivery efficacy [22,23,24]. Recently, nanocarriers have received great attention because of their biocompatibility and capability to enhance drug stability and prevent the drug from rapid degradation.

Metal-organic frameworks (MOFs) are coordination polymers with porous crystalline forms developed when metal ions are coordinated to an organic cross-linker. MOFs are based on the chemistry of metal carboxylate clusters, in which the metal and the organic carboxylate linker form a three-dimensional rigid framework [25]. They are of considerable interest to organic and inorganic chemists and materials scientists due to their extraordinarily large surface area, endless tenability, stability, excellent porosity, and well-defined crystalline structures [26,27]. Because of their utilization in a variety of applications, they are considered an exciting material in the current science [28]. Due to their composition, structure, and interior surface volume, MOFs can act as a reservoir for antimicrobials agents. These properties make MOFs an attractive candidate for utilization as a new class of high-performance materials with antibacterial activities [29].

Carbohydrates are cheap and readily available, and it is well-known that sugar chemistry allows their efficient modification and characterization. Carbohydrates play an essential function in the living body since they are involved in a variety of biological processes, including the differentiation and development of living organisms, as well as pathological activities [30,31]. The surface of a mammalian cell is covered with a dense layer of carbohydrate coating known as glycocalyx [32]. It exists in both bacterial and eukaryotic cells in the form of proteoglycans, glycans, glycolipids, or glycoproteins, and it is involved in communication and cell–cell interaction, as well as signal transduction. Additionally, they play a key role in cell development and a variety of immunological responses [33,34].

Several pathogens are capable of binding to the carbohydrate on the cell surface and causing infection [35,36]. Carbohydrates, in general, exert a strong influence on the process of cell surface adhesion through carbohydrate–receptor (lectin) or carbohydrate–carbohydrate interactions [34,37]. Most carbohydrate molecules (e.g., galactose, lactose, MNS) are used to bind with lectin receptors commonly found in liver cells and also on the bacterial cell surface [38]. MNS-coated MOFs were used to enhance the bactericidal activity of VCM against *Staphylococcus aureus*-resistant strains in this study. MNS coating on the MOFs surface enhances the adherence of MOFs on the bacteria by binding with lectins found on the bacterial cell and leads to the cellular penetration of VCM with increased activity against resistant bacteria.

## 2. Materials and Methods

Iron (III) chloride hexahydrate (FeCl_3_·6H_2_O, 99%) was purchased from Riedel-de Häen (Seelze, Germany). Poly-lysine, 2-aminoterephthalic acid (H_2_N-BDC), 3-(4, 5-dimethylthiazol-2-yl)-2,5-diphenyltetrazolium bromide (MTT), and glutaraldehyde were obtained from Sigma Aldrich (Darmstadt, Germany). Mueller Hinton broth and Tryptic soy agar were purchased from Oxoid, Basingstoke, UK. Bosch Pharmaceuticals (Pvt) Ltd., Pakistan, generously provided VCM-HCl. All solvents employed in this investigation were of analytical quality and were not purified further.

Fe-MIL-88NH_2_ was prepared according to the previously reported protocol [39]. Briefly, FeCl_3_·6H_2_O (0.187 g; 0.692 mmol) and 2-aminoterephthalicacid (0.126 g; 0.692 mmol) were dissolved in 15 mL of DMF followed by the addition of 3.45 mmol of acetic acid into the resulting mixture. The mixture was then placed in a hot air sterilizer for 4 h to form the crystals. After that, the excess reactants were removed by washing with DMF and ethanol, and pure crystals were obtained by centrifugation. Finally, the Fe-MIL-88NH 2 MOF was dried under a vacuum oven.

For the encapsulation of VCM into the synthesized Fe-MIL-88NH_2_, 30 mg of each (VCM and Fe-MIL-88NH_2_) was mixed in distilled water (10 mL) and allowed to stir at 200 rpm for 24 h. The mixture was then centrifuged for 15 min at 12,000 rpm to obtain VCM-loaded Fe-MIL-88NH_2_ (VCM-Fe-MIL-88NH_2_). The supernatant was discarded, and the pellet was allowed to release its loaded drug in water by applying the sonication for 20 min. The mixture was centrifuged at 12,000 rpm for 15 min and the amount of released VCM was determined at 280 nm by UV-Vis spectrophotometry (UV-240, Shimadzu, Kyoto, Japan).

The following formula was used to determine the encapsulation efficiency (%EE):

$$\%\mathrm{EE}=\frac{\mathrm{Amount}\;\mathrm{of}\;\mathrm{VCM}\;\mathrm{loaded}\;\times 100}{\mathrm{Total}\;\mathrm{amount}\;\mathrm{of}\;\mathrm{VCM}\;\mathrm{used}}$$

VCM-Fe-MIL-88NH_2_ MOF was further functionalized with Mannose (MNS). Briefly, a 0.238 mM solution of MNS was prepared in water, which was added dropwise to the citrate buffer (pH 4.0) containing the same millimoles of VCM-Fe-MIL-88NH_2_ MOF, and the resulting mixture was stirred for 2 h at 55 °C. Following that, NaBH_4_ (20 eq.) was added to the reaction mixture, which was then stirred for 3 days at 55 °C. The product was separated after centrifugation at 12,000 rpm for 15 min.

### 2.1. Thermogravimetric Analysis (TGA) and Differential Scanning Calorimetry (DSC)

The thermal properties of prepared samples were investigated through TGA and DSC. TGA analysis was performed on TA instruments SDT Q600, and the samples (6 mg) were heated at a rate of 10 °C/min at 50 kPa pressures in a nitrogen environment between 25 °C and 700 °C. DSC analysis was conducted by following the same procedure and instrument used for TGA.

Powder X-ray diffraction (P-XRD) was utilized to characterize the crystalline structure, size, and purity of synthesized Fe-MIL-88NH_2_. Diffraction patterns were assessed employing an XRD instrument (Axios Petro, PANalytical, CoKα, λ = 1.79021 Å) from 5° to 30° (2θ) with Cu-Kα irradiation.

### 2.2. Surface Morphology

A dynamic light scattering (DLS) instrument (Nano ZS90 Malvern Instruments, Worcestershire, UK) was used to determine the PDI, the size, and the zeta-potential. The samples for DLS were dispersed in water and evaluated in triplicate on DLS at 25 °C with a scattering angle of 90°. An atomic force microscope (AFM, 5500, Agilent, Santa Clara, CA, USA) was used to examine the morphology of the synthesized MOFs. The diluted samples were put onto a mica slide, allowed to dry at room temperature, and then examined under a microscope.

To analyze the possible interaction of VCM and MNS with MOFs, FT-IR analysis was performed. The minimum sample was ground with KBr to form an amorphous mixture, which was subsequently transformed into a translucent pallet using 200 psi pressure. The samples were analyzed in the range of 400–4000 cm^−1^. 

### 2.3. Atomic Force Microscopy (AFM) Analysis

The surface morphology of prepared Fe-MIL-88NH_2_ was studied through AFM. AFM analysis was performed using an atomic force microscope (5500, Agilent, Santa Clara, CA, USA). Colloidal solution of Fe-MIL-88NH_2_ was placed on mica slides, air-dried, and examined under the microscope. Images were recorded in non-contact mode. 

### 2.4. Antibacterial Assay

Antibacterial assay was carried out on Gram-positive sensitive bacterial strains which included sensitive strain (*Staphylococcus aureus* ATCC 6385) and resistant strains (*Staphylococcus aureus* ATCC 700699, and clinical isolate of *Staphylococcus aureus*). Bacterial strains were cultured on tryptic soy agar (Oxoid, Basingstoke, UK) at 4 °C. Before the antibacterial test, all of the microbial strains were subcultured for 24 h on a fresh suitable agar plate.

The inocula were prepared by introducing multiple single colonies of microorganisms to a sterilized Mueller Hinton broth. The bacterial cell solution was homogeneously mixed to a final density of 5 × 10^5^ CFU/mL, which was confirmed with viable counts. Most of the microorganisms have an infective dosage of 10^5^ CFU/mL.

### 2.5. Minimum Inhibitory Concentration Assay with Tetrazolium Microplates

The tetrazolium microplate assay was employed to analyze the minimum inhibitory concentration (MIC) of the experimental sample and reference substances [40]. A 96-well clear microtiter plate was used for experiments. Each well was inoculated with freshly obtained *Staphylococcus aureus* (clinical, Sensitive, and resistant species) cell suspensions at a concentration of 5 × 10^5^ CFU/mL. Different concentrations from 250 to 10 µg of VCM, Fe-MIL-88NH_2_, VCM-Fe-MIL-88NH_2_, and MNS-VCM-Fe-MIL-88NH_2_ were prepared in Muller Hinton broth, and then 200 µL of each concentration was put in triplicate wells and the plate was incubated at 37 °C ± 0.5 for 18–24 h. Following incubation, 50 µL of 3-(4, 5-dimethylthiazol-2-yl)-2, 5-diphenyltetrazolium bromide MTT (0.2 mg/mL) was added to each well and the plate was incubated for 30 min at 37 °C. A suitable solvent blank (DMSO) was used as a negative control, while a bacterial suspension was used as a positive control. By introducing DMSO to a spectrophotometer, the absorbance was set at 570 nm and measured with a reference wavelength of 650 nm. Then, the percentage reduction in the dye (showing bacterial growth suppression) was computed as [41].

### 2.6. Analyses of the Surface Morphology

*Staphylococcus aureus* (sensitive and resistant) strains were harvested up to 24 h at 37 °C in TSA. Poly-lysine was loaded on a mica slide and dried at room temperature followed by loading of the bacterial strain of density 10^5^ CFU/mL, dried, and analyzed using AFM. The strains treated with test samples were used for their comparative study using the same protocol. At the MIC, around 10 μL of each sample was loaded on a mica slide containing poly-lysin and dried at room temperature, then analyzed under the microscope.

### 2.7. Statistical Analysis

All experiments were conducted in triplicate, and the results were recorded as mean ± SEM.

## 3. Results

### 3.1. FT-IR Analysis

**Figure 1:**
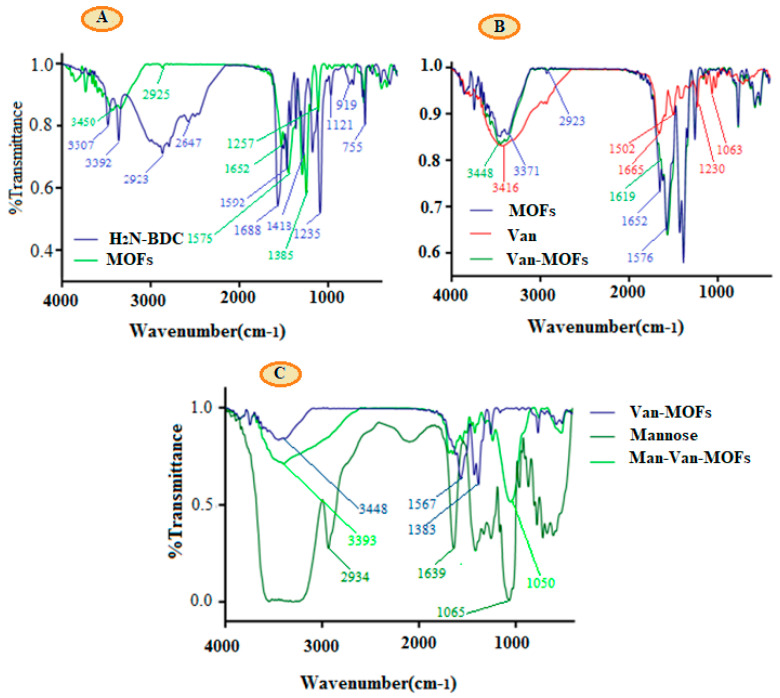
FTIR spectra of NH_2_−BDC and MOFs (**A**), MOFs, VCM and VCM−MOFs (**B**). MNS, VCM−MOFs and MNS−VCM−MOFs (**C**).

To confirm the synthesis of Fe-MIL-88NH_2_, VCM loading, and surface modification of Fe-MIL-88NH_2_ by MNS, FT-IR analysis was used. Figure 1A illustrates the FTIR spectra of NH_2_-BDC and Fe-MIL-88NH_2_. NH_2_-BDC spectrum showed characteristic peaks at 3507 and 1688 cm^−1^, representing O–H and C=O stretching, respectively, of the acid group, while peaks at 3392 cm^−1^ and 1592 cm^−1^ were related to the primary amines [42]. The FT-IR spectrum of Fe-MIL-88NH_2_ revealed a characteristic peak at 1652 cm^−1^ and 2923 cm^−1^, representing C=O and C–H stretching, respectively [43]. The NH_2_ stretching and bending frequencies appear at 3371 and 1576 cm^−1^, respectively [42]. The vibrational band at 1385 and 1576 cm^−1^ was attributed to asymmetric and symmetric stretching of the metal coordinated carboxylic groups of the NH_2_-BDC. The absence of typical peaks that correspond to protonated carboxylic groups of NH_2_-BDC in the region of 1715–1680 cm^−1^ indicated the complete deprotonation of amino-terephthalic acid in Fe-MIL-NH_2_ [44,45]. In the spectrum of VCM, the absorption bands at 3416, 1665, and 1230 cm^−1^ represent hydrogen-bonded OH stretching, C=O, and phenolic OH stretching, respectively. These characteristic peaks of VCM also appeared in VCM-loaded Fe-MIL-88NH_2_ [46,47]. The characteristic peak of MNS appeared in the region of 3600–3200 cm^−1^, showing O-H stretching, while C-O-C absorption appeared at 1065 cm^−1^ [48]. After coating MNS, the VCM-MOFs peaks appeared at 1567 cm^−1^ and 1383 cm^−1^, representing that NH stretching disappeared, showing that the respective functional groups are involved in interaction with MNS, while the characteristic C-O-C linkage of MNS appeared at 1050 cm^−1^ with a new characteristic peak at 1553 cm^−1^ for newly formed C=N bond.

Summarized characteristic peaks of NH_2_BDC, MOFs, VCM, VCM-MOFs, MNS, and MNS-VCM-MOFs are presented in Table 1.

### 3.2. Size, PDI, Zeta-Potential, and Surface Morphology

The PDI, zeta potential and size were all determined using the DLS approach. The size of the particles in drug delivery dosage forms is a critical aspect since it influences both the physical stability and medicinal effectiveness of the loaded drugs. MOFs are porous crystalline nanomaterials, and the preparation method as well as the utilization of solvent in their synthesis have a significant impact on their particle size. Water and methanol produce larger particles than DMF. This is because the cross-linkers used to make MOFs are more soluble in DMF [49]. As indicated in Table 2, the particle size of MOFs was 490.40 ± 0.63 nm with a PDI of 0.61 ± 0.02, which was increased to 564.03 ± 37.69 and 683.36 ± 21.42 nm with PDI values of 0.66 ± 0.04 and 0.68 ± 0.02, and zeta potential of −15 ± 0.50 and −20 ± 1.30, respectively, during drug loading and surface modification.

**Figure 2:**
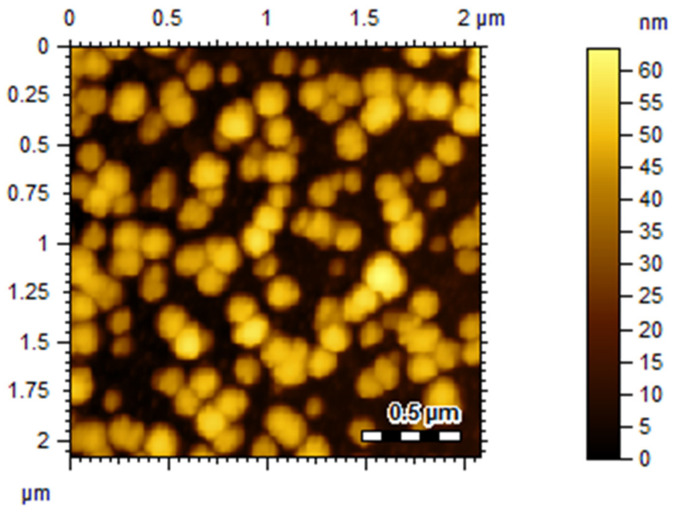
Surface morphology of VCM-MOFs.

VCM-MOFs have a spherical structure, as demonstrated by morphological examination (Figure 2).

### 3.3. Drug Loading Efficiency

The developed delivery system’s efficacy in loading therapeutic compounds ensures their unloading at the target sites, resulting in increased therapeutic efficacy. MOF loading efficiency is directly connected to its surface area, regular and massive cages, and tunnels, as the drug is loaded in MOF cages and tunnels [50]. As reported in Table 2, the loaded content of VCM in synthesized MOFs was 70.87 ± 2.65. The modification of MOFs with MNS resulted in a slight decrease in drug loading efficiency to 65.21 ± 4.15.

### 3.4. Thermogravimetric (TG) and Differential Scanning Calorimetry (DSC) Thermal Analysis

**Figure 3:**
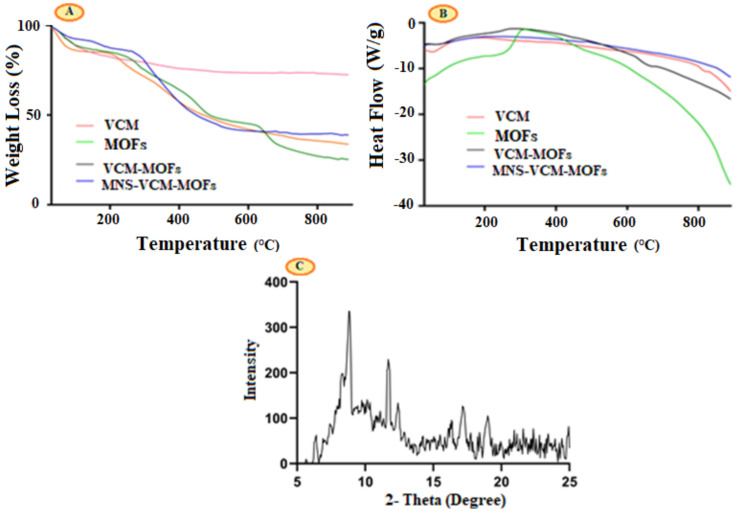
TG curve of VCM, MOFs, VCM−MOFs, and MNS−VCM−MOFs (**A**), DSC diagram of VCM, MOFs, VCM−MOFs, and MNS−VCM−MOFs (**B**), and P−XRD pattern (**C**).

The TGA curve of each sample is given in Figure 3. Almost the same thermal stability was observed for the VCM, MOFs, and VCM-MOFs, while greater stability was only observed for MNS-VCM-MOFs. VCM revealed the weight loss in three different stages, i.e., 42.12 °C and 284.33 °C, corresponding to 3.8% and 26% weight loss, respectively, while 50% loss was observed at a higher temperature of 478.79 °C. 

After encapsulation in MOFs, the stability of VCM slightly changed, i.e., the first weight loss was increased to 90.03 °C for 9.931% weight loss, while 30% weight loss was observed at 345.92 °C and 50.07% at 488.94 °C. MNS-VCM-MOFs showed 24.92% weight loss at higher temperatures than VCM-MOFs, i.e., at 456.21 °C with 17.47% loss at 195.60 °C, as shown in Figure 3A. This indicated that when VCM-MOFs were functionalized with MNS, their stability was increased. Percent weight loss below 200 °C can be attributed to the dehydration process, whereas weight loss between 350 and 500 °C can be related to the removal of organic links in the network.

DSC analysis was performed to examine the physical properties of VCM in synthesized samples. A DSC thermogram of VCM revealed a single endothermic peak at 279.48 °C, indicating its crystalline character and melting point. VCM-MOFs and MNS-VCM-MOFs did not exhibit a peak over the specified temperature range, as illustrated in Figure 3B. This VCM can be found in its crystalline form inside MOFs and remained intact after inclusion in synthesized MOFs.

### 3.5. Powder-XRD

The phase purity and crystalline nature of prepared Fe-MIL-88NH_2_ were studied using P-XRD. The powder XRD pattern is shown in Figure 3C where main crystalline peaks were observed at 9.05°, 10.6°,16.9°,17.7°, and 18.9°, which showed that the crystal structure was similar to that reported in a previous study [36].

### 3.6. Anti-Bacterial Assay

#### 3.6.1. Tetrazolium Microplate Assay

**Figure 4:**
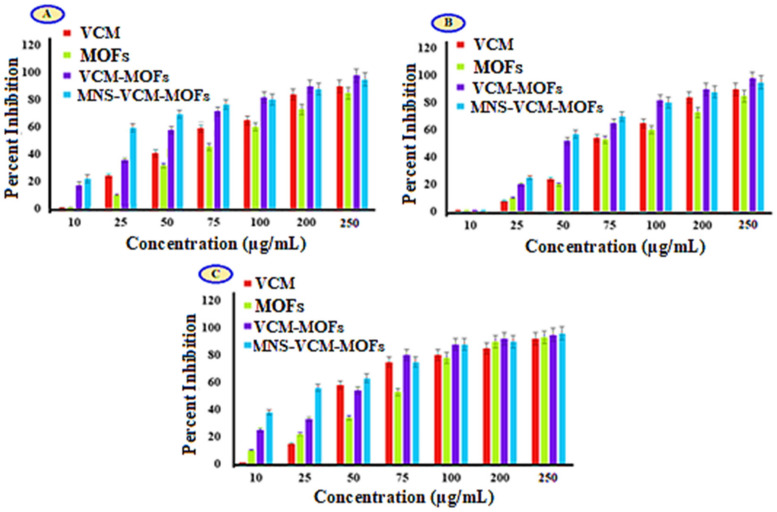
MIC of *Staphylococcus aureus* ATCC 6538 (**A**), VRSA ATCC 700,699 (**B**), and *Staphylococcus aureus* clinical isolate (**C**).

MIC value for all the samples determined via tetrazolium microplate assay and results are summarized in Figure 4. The results revealed that the MIC value of VCM against sensitive strains (*Staphylococcus aureus* ATCC 6538) is 25 µg/mL with 30 ± 4.0% inhibition of bacterial growth, while it was reduced upon encapsulation in MOFs and functionalized MOFs, i.e., 10 µg/mL with 22.0 ± 3.0% and 32.4 ± 4.0% (Figure 4A). For the case of resistant *Staphylococcus aureus* strains (VRSA ATCC 700699), the MIC of VCM was found to be 25 µg/mL with 08 ± 0.3%. After encapsulation in MOFs, the percent inhibition increased to 20 ± 0.2% (VCM-MOFs) and further increased to 25 ± 0.3% after encapsulation in MNS-coated MOFs (MNS-VCM-MOFs) (Figure 4B).

The potent activity was observed for the *Staphylococcus aureus* clinical isolate, i.e., the MIC value of VCM was reduced to 10 µg/mL with 25 ± 0.3% and 38 ± 0.3% for VCM-MOFs and MNS-VCM-MOFs, respectively (Figure 4C).

#### 3.6.2. Morphological Studies

**Figure 5:**
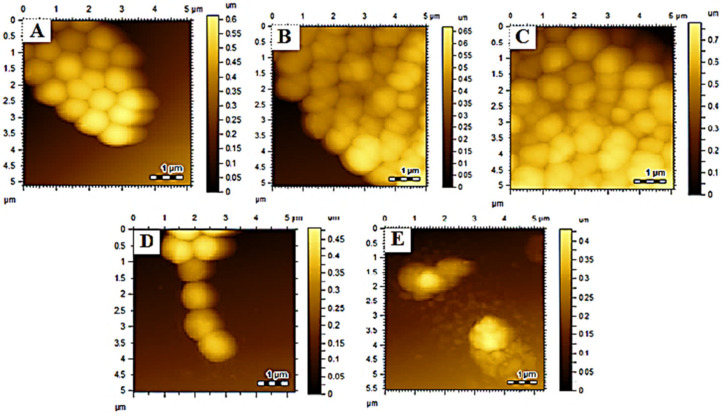
A Surface morphological analysis of *S. aureus* (sensitive) control (**A**), VCM treated (**B**), MOFs treated and (**C**), VCM−MOFs treated (**D**) and MNS−VCM−MOFs treated (**E**).

Morphological studies are beneficial to investigate the effect of nanoparticles and drug delivery systems on bacterial cells [15,51]. The bactericidal activity was further confirmed by morphological analysis using AFM. Bacterial morphology was determined after treatment with samples, i.e., VCM, MOFs, VCM-MOFs, and MNS-VCM-MOFs, and the results are summarized in Figure 5. Treatment with VCM, MOFs, and VCM-MOFs did not reveal significant damage on *Staphylococcus aureus* ATCC 6538 strains, as shown in Figure 5B–D. While MNS-VCM-MOFs were destroyed, the morphology of bacterial strains and cells appeared as a melted material (Figure 5E).

**Figure 6:**
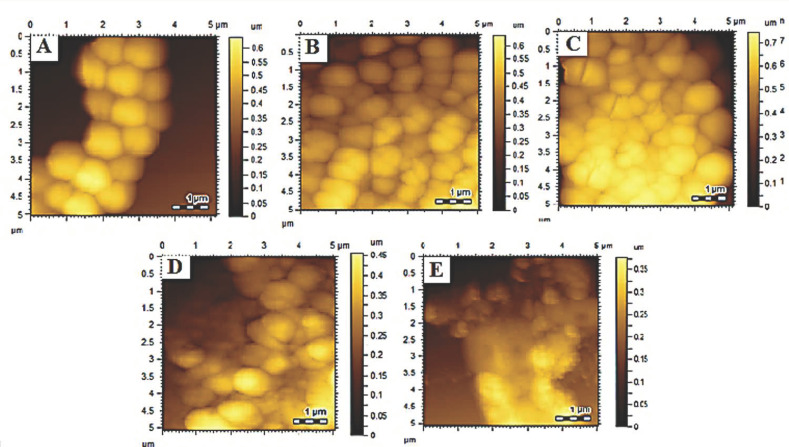
A surface morphological analysis of *Staphylococcus aureus* (VRSA) control (**A**), VCM treated (**B**), MOFs treated (**C**), VCM−MOFs treated (**D**), and MNS−VCM−MOFs treated (**E**).

Similarly, the resistant *S. aureus* retained its cellular morphology after treatment with VCM and MOFs (Figure 6B,C, respectively) but slight damage was observed upon treatment with VCM-MOFs (Figure 6D). The potent effect was observed for the strains treated with MNS-VCM-MOFs as they completely disintegrated the bacterial cells (Figure 6E). 

**Figure 7:**
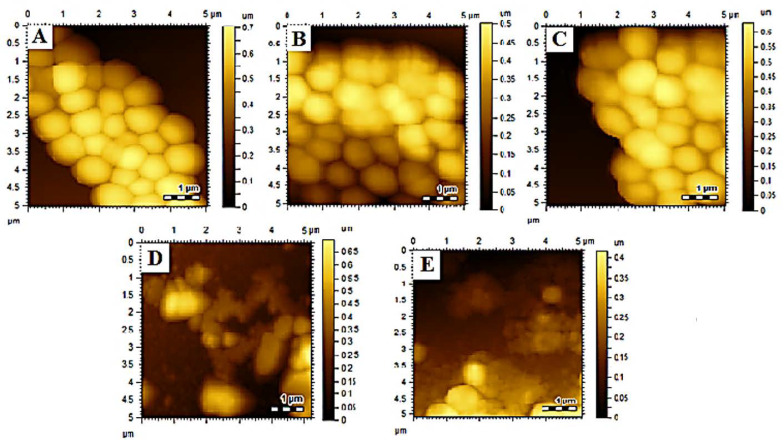
A surface morphological analysis of *Staphylococcus aureus* (clinical isolate) control after incubation (**A**), VCM treated (**B**), Fe−MIL−88NH_2_ treated and (**C**), VCM−Fe−MIL−88NH_2_ treated (**D**), and MNS-VCM−Fe−MIL−88NH_2_ treated (**E**).

The clinical isolate of *S. aureus* was similarly unaffected by VCM and MOFs treatment (Figure 7B,C, respectively), but the cellular morphology was damaged and completed upon treatment with VCM-MOFs and MNS-VCM-MOFs (Figure 7D,E, respectively).

## 4. Discussion

Bacterial infections present a significant challenge to clinical therapy due to the development of antibiotic resistance [52]. Gram-positive bacteria, such as MRSA, have become the leading causes of chronic skin infections and pneumonia because of their decreased susceptibility to vancomycin. Consequently, conventional agents based on antibiotics are no longer able to effectively control infection. To overcome this serious issue, various nanomaterials of noble metals [53] and semiconductors [54] have been designed to solve this problem. Metal–organic frameworks (MOFs), which are assembled from inorganic metal ions or clusters and organic ligands into periodic networks, are one of the alternative nanomaterial candidates for use in antibacterial applications [55] and have attracted rising attention during recent years.

Here, Fe-MIL-88NH_2_ was successfully synthesized after VCM was encapsulated in the synthesized MOFs. The MOFs were then functionalized by MNS to enhance the antibacterial potential of encapsulated VCM. The interaction of VCM with synthesized MOFs was characterized by FT-IR analysis. The result reveals that the characteristic peaks of VCM appeared in the MOFs with slight variations. Similarly, the characteristic peaks of MNS were also observed in the synthesized MNS-VCM-MOFs with slight modification. The PDI, zeta potential and size were all determined using the DLS approach. It was observed that the VCM-MOFs and MNS-VCM-MOFs have a relatively greater size, which may be related to surface modification and drug loading with MNS.

After MNS coating and drug loading, the polydispersity of the prepared MOFs increased. This can be related to the MOFs having unequal drug loading and being coated with MNS. Another significant aspect of the drug delivery carrier is the zeta potential, which refers to the overall net charge of the suspended particles. All synthesized MOFs have a negative zeta potential due to the presence of different hydroxyl moieties in MNS and carboxylic groups in the crosslinker. Coating MOFs with MNS resulted in an increase in the negative zeta potential to −20 ± 1.30 mV. This higher zeta potential was expected to provide physical and storage stability on the MOFs, as particles with increased identical charges resist one another and remain suspended. Additionally, the drug remains intact in MOFs due to their MNS-coated surfaces. Furthermore, morphological analysis results revealed that the synthesized VCM-MOFs had a spherical shape. The drug loading efficiency of MOFs was determined to be 70.87 ± 2.65%; this could be a result of drug trapping in their pores, noncovalent interactions (pi–pi stacking and hydrogen bonding), and probable formation of dative bonds with electron-deficient metals in the surrounding environment. The drug loading efficiency of MNS-coated MOFs was decreased to 65.21 ± 4.15. This could be due to MNS coating displacing or removing surface loosely attached drug molecules.

The World Health Organization (WHO) suggests that the chemical and thermal stability of products be evaluated to identify any degradation species in final medicinal products. Understanding the response of drugs, excipients, and their formulations to thermal stresses is, therefore, unquestionably essential to the development of pharmaceutical products. The thermal stability of medications is a matter of significant pharmaceutical importance [56]. Therefore, the thermal stability of synthesized MOFs was determined using TGA analysis. The result of the analysis revealed that when VCM was encapsulated in MNS-coated MOFs, its stability was highly enhanced so that only 24.92% weight loss was observed at 456.21 °C. DSC was used to ascertain the VCM’s physical characteristics in the synthesized MOFs. From the inside of drug delivery systems, DSC studies confirm whether the drug is amorphous or crystalline. Furthermore, changes in the endothermic peaks can be used as a predictor of the degree of crystallinity of the drug. In this study, no shift was observed in the endothermic peak of VCN which indicated that the loaded VCN was not susceptible to being affected by temperature and remained intact in its crystalline form inside the pores of synthesized MOFs. Powder XRD is the most effective technique for elucidating the structure of nanomaterials. XRD patterns can reveal an abundance of information, from phase composition to crystallite size, and from lattice strain to crystallographic orientation [57]. The main crystalline peaks of the prepared Fe-MIL-88NH_2_ were observed at 9.05°, 10.6°, 16.9°, 17.7°, and 18.9°, which revealed that the crystal structure of the prepared MOFs was almost consistent with the structure, as reported previously in the literature.

The antibacterial activity of synthesized MOFs was determined in terms of MIC values against sensitive, resistant, and clinically isolated strains of S. aureus. The results revealed that the MIC value of VCM was significantly reduced from 25 µg/mL with 30 ± 4.0% inhibition to 10 µg/mL with 32.4 ±4.0% inhibition after encapsulation in MNS coated MOFs for sensitive strain, while in the case of the resistant strain, the % inhibition of VCM was increased from 08 ± 0.3% to 25 ± 0.3% after encapsulation in MNS-MOFs. Similarly, the % inhibition for the clinically isolated strain was determined to be 38 ± 0.3% at 10 µg/mL. The results suggested that the bactericidal activity of VCM significantly increases in MNS functionalized VCM-loaded MOFs; this may be because of the interaction of MNS with bacterial lectins, which eventually enhances the bactericidal potential of VCM [58].

Morphological studies are beneficial to investigate the effect of nanoparticles and drug delivery systems on bacterial cells [15,58]. The antibacterial activity of the synthesized MOFs was further confirmed by morphological investigation through AFM. The result revealed that the bacterial cell was destroyed after treatment with MNC-coated MOFs for all of the strains investigated in the current study, demonstrating the enhanced antibacterial potential of VCM in loaded in synthesized MNS-coated MOFs.

## 5. Conclusions

Here, we successfully synthesized the MNS-functionalized Fe-MIL-88NH_2_, MOFs by solvothermal method with 65.21 ± 4.15% encapsulation efficiency for VCM. The synthesized drug-loaded MOFs were found to be highly stable against temperature and exhibited strong bactericidal activity of the MNS-VCM-Fe-MIL-88NH_2_ against *Staphylococcus aureus* (sensitive, resistant, and clinical species), which were confirmed by AFM images.

The study suggests that MNS-functionalized MOFs increased the bactericidal efficacy of VCM by destabilizing the effect on bacterial surface morphology.

## Figures and Tables

**Table 1:** FT-IR assignment of NH_2_-BDC, MOFs, VCM, VCM-MOFs, MNS, and MNS-VCM-MOFs.

FT-IR Peaks (cm^−1^)						
NH_2_-BDC	MOFs	VCM	VCM-MOFs	Mannose	MNS-VCM-MOFs	Vibrational Mode
3707	-	3416	3448	3600–3200	3393	-OH stretching
3392	3371	-	-	-	-	-NH_2_ stretching
	2923	-	-	2934	-	-CH
1688	1652	1665	1619	-	-	-C=O
1592	1576	-	-	-	Disappeared	-NH_2_
		1230	1383	1065	1050	-C-O
-	1385 and 1576	-	-	-	Disappeared	Metal coordinated -COOH

**Table 2:** Size, zeta-potential, PDI, and drug loading efficiency (DLE) characterization.

Test Samples	Size (nm)	PDI	Zeta Potential (mV)	%EE
MOFs	490.40 ± 0.63	0.61 ± 0.02	−12.4 ± 2.94	
VCM-MOFs	564.03 ± 18.47	0.66 ± 0.04	−15 ± 0.50	70.87 ± 2.65
MNSVCMMOFs	683.36 ± 21.42	0.68 ± 0.02	−20 ± 1.30	65.21 ± 4.15

## Data Availability

The data supporting this study are available from the corresponding author upon reasonable request.
